# TrhOnt: building an ontology to assist rehabilitation processes

**DOI:** 10.1186/s13326-016-0104-y

**Published:** 2016-10-04

**Authors:** Idoia Berges, David Antón, Jesús Bermúdez, Alfredo Goñi, Arantza Illarramendi

**Affiliations:** University of the Basque Country, UPV/EHU, Paseo Manuel de Lardizabal, 1, Donostia-San Sebastián, 20018 Spain

**Keywords:** Ontologies, Knowledge representation, Clinical decision support systems in physiotherapy

## Abstract

**Background:**

One of the current research efforts in the area of biomedicine is the representation of knowledge in a structured way so that reasoning can be performed on it. More precisely, in the field of physiotherapy, information such as the physiotherapy record of a patient or treatment protocols for specific disorders must be adequately modeled, because they play a relevant role in the management of the evolutionary recovery process of a patient. In this scenario, we introduce TrhOnt, an application ontology that can assist physiotherapists in the management of the patients’ evolution via reasoning supported by semantic technology.

**Methods:**

The ontology was developed following the NeOn Methodology. It integrates knowledge from ontological (e.g. FMA ontology) and non-ontological resources (e.g. a database of movements, exercises and treatment protocols) as well as additional physiotherapy-related knowledge.

**Results:**

We demonstrate how the ontology fulfills the purpose of providing a reference model for the representation of the physiotherapy-related information that is needed for the whole physiotherapy treatment of patients, since they step for the first time into the physiotherapist’s office, until they are discharged. More specifically, we present the results for each of the intended uses of the ontology listed in the document that specifies its requirements, and show how TrhOnt can answer the competency questions defined within that document. Moreover, we detail the main steps of the process followed to build the TrhOnt ontology in order to facilitate its reproducibility in a similar context. Finally, we show an evaluation of the ontology from different perspectives.

**Conclusions:**

TrhOnt has achieved the purpose of allowing for a reasoning process that changes over time according to the patient’s state and performance.

## Background

Whenever a patient is treated in a physiotherapy unit some amount of information is generated, which includes the clinical data relevant to the current situation of the patient, as well as information regarding their personal habits and family history. This information composes the physiotherapy record of a patient and must be adequately modeled in order to be efficiently consulted. Moreover, it is important to recognize achievements of goals in order to manage the evolutionary recovery process of the patient. For that reason, specific protocols must be defined for specific disorders and customization of exercises is usually needed depending on the circumstances of each patient. Thus, knowledge about state and context of patients, disorders, phases of protocols, goals, achievements, and recommended or contraindicated exercises depending on the patients’ state must be properly represented to assist the design of the treatments and to support some decisions during their execution.

Undoubtedly, information technologies are playing a relevant role in the research and improvement of the healthcare domain [1]. Proof of this fact is the plethora of works that have been published in this area. Since the purpose of this paper is to present an ontology for physiotherapy, we will restrict the review of the related literature to three kinds of works: (1) works which address the development of ontologies for different areas related to medicine other than physiotherapy; (2) works that focus on the field of physiotherapy but use technologies other than ontologies; (3) works where ontologies for physiotherapy are presented.

Many solutions in the field of medicine [2–5] tend towards the use of semantic technologies such as ontologies, which can play a relevant role in transforming information into knowledge that facilitates the work of the physicians. Thus, a great effort has been made on the development of ontologies that cover medical knowledge [6]. One example is the Foundational Model of Anatomy (FMA) [7], whose current release contains over 100,000 classes and properties for the OWL representation of the phenotypic structure of the human body. Ontologies have also been used for Clinical Decision Support (CDS) [8] in health-related fields. In [9] an ontology for cardiac intensive care units is introduced, which captures the patient’s vital parameters and provides experts with a recommendation regarding the treatment to be administered. In [10] an ontology-based pervasive healthcare solution is presented with the purpose of delivering e-health services in self care homes, such as recommendations about daily physical activities, changes of room temperature, etc. due to the resident’s conditions. The ontology developed in [11] uses both recent information taken at the point of care and past patient data stored in electronic health records to provide support to three clinical applications: triage of pediatric abdominal pain, triage of pediatric scrotal pain and postoperative management of radical prostatectomy. In [12] ontologies are used in the development of a preoperative assessment system to recommend preoperative tests to patients while in [13] a lung cancer ontology for categorizing patients and producing guideline-based treatment recommendations is described.

Considering the field of physiotherapy, software for physiotherapy and rehabilitation managing has been available, on the one hand, as commercial products for some years [14, 15]. These systems represent the transition from a paper-based storage to standardized electronic records, but while they have been specially focused on data recording and administrative purposes, they do not use technologies such as CDS that would allow them to deepen in the use of the data gathered for assisting physiotherapists in diagnosis, treatment definition and patient monitoring. On the other hand, there exist proposals such as Gross et al. [16] that use machine learning techniques to develop a CDS tool for selecting appropriate rehabilitation interventions for injured workers; Hawamdeh et al. [17] that use resilient backpropagation artificial neural network algorithm to accurately predict the rehabilitation protocols prescribed by the physicians for patients with knee osteoarthritis; and finally, Farmer [18], which presents a CDS system based on a Bayesian belief network for musculoskeletal disorders of the shoulder.

However, to the best of our knowledge, only few physiotherapy-related works address problems from the point of view of semantic technologies. Button et al. [19] present TRAK, an ontology that models information for the rehabilitation of knee conditions. It aims to standardize knee rehabilitation terminology and integrate it with other relevant knowledge sources. Although we acknowledge the usefulness of TRAK, we feel that it does not take advantage of all the capabilities that semantic technologies offer, especially with regard to reasoning, which would require greater detail in the definition of concepts. In [20] Dogmus et al. introduce REHABROBO-ONTO, an ontology that represents information about rehabilitation robots. This ontology helps in the process of selecting the right rehabilitation robots for a particular patient or a physical therapy, by means of a web interface. However, this solution does not integrate the description of the patient report and thus it is just a query tool.

In this paper we present TRHONT (Telerehabilitation Ontology), an ontology whose goal is to assist physiotherapists in the following daily tasks:

- *Recording and searching information about the items that compose the physiotherapy record of a patient*: providing a means to represent information regarding age, symptoms, personal and family history, recovery goals, results of explorations, etc. in a structured way allows for reasoning about that information. Notice that it also facilitates interoperability with Electronic Health Record (EHR) data recorded in other institutions which also make use of ontologies [21].
- *Defining treatment protocols for a specific disorder, by selecting the exercises that can be performed in each phase of the protocol*: a treatment protocol is composed usually of different phases that a patient must go through until their recovery is completed. Each of the phases contains exercises whose difficulty level is in line with one that the phase requires. Among others, the representation of protocols, phases, and exercises in an ontology allows for a reasoning-based selection of suitable exercises for each phase. Moreover, definitions of new protocols, phases or exercises can be proven consistent by means of reasoning.
- *Identifying in which phase of a treatment protocol a patient is at some specific moment*: resoning plays a relevant role in the decision making process related to the evolution of the patient. Thanks to the ontological description of the state of the patient, and more precisely of the results that they have achieved when performing the exercises, the patient can be classified in one phase of the treatment protocol. However notice that this classification is not final: it will evolve alongside the evolution of the patient in the therapy.
- *Identifying which exercises are most suitable for a patient at some specific moment*: given all the information that is known about a patient (their current state, their personal history, their age, etc.) the ontology provides a means to identify which of the exercises are recommended or contraindicated for them at that specific moment. As a result, the most suitable exercises for the patient can be detected and suggested.

The contribution presented in this paper focuses on the rehabilitation of the glenohumeral joint. Nevertheless, the general nature of our method makes it reproducible to model any other body structure deserving rehabilitation.

## Methods

In order to achieve the goal described in the previous section we implemented an ontology following the NeOn methodology [22]. The NeOn Methodology framework presents a set of scenarios for building ontologies and ontology networks. These scenarios are decomposed into several processes or activities, and can be combined in flexible ways to achieve the expected goal.

**Fig. 1:**
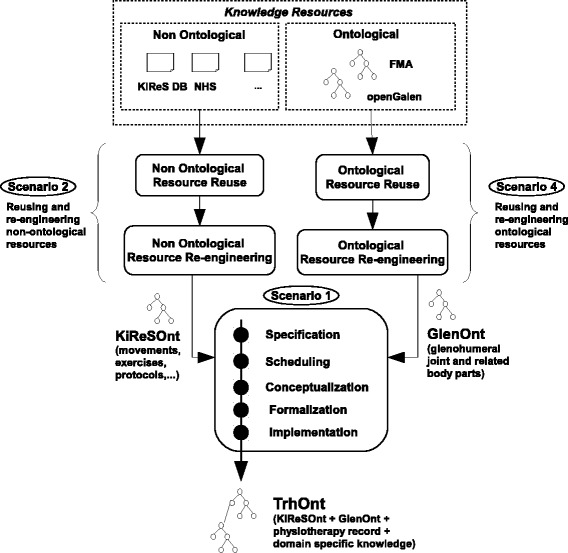
Scenarios of NEON used in the development of TRHONT. Scenarios for building ontologies and ontology networks that were used in the development of TRHONT. Adapted from [22]
- Movements, exercises and treatment protocols: An ontology module named KiReSOnt (Kinect Rehabilitation System Ontology) was developed. This module is imported by TrhOnt.
- A description of a selected part of the human body: We focused on the glenohumeral joint and the body parts that are related to it. An ontology module named GlenOnt (Glenohumeral Ontology) was developed. This module is also imported by TrhOnt.
- Other relevant information for the physiotherapeutic domain.

In our case three of the scenarios proposed by NeOn (scenarios 1, 2 and 4, see Fig. 1) have been combined to obtain the current version of the ontology, named TRHONT, which contains over 2400 classes and properties to represent:

- The physiotherapy record of a patient.

A detailed account of how each of those scenarios was applied is presented next.

### Scenario 1: from specification to implementation

This scenario is composed of the five core activities to be performed in the development of any ontology: ontology requirements specification, scheduling, conceptualization, formalization and implementation.

- *Ontology requirements specification:* It produces as output the Ontology Requirements Specification Document (ORSD), where information such as the purpose, the scope and the intended uses of the ontology is described (Table 1). Special attention must be paid to the definition of groups of competency questions, which are the set of questions that the ontology must be able to answer. In our case, competency questions related with physiotherapy records, body parts and treatment protocols were defined, as well as some general-purpose competency questions that either fall in more than one of those categories or do not fall in any of them.

**Table 1:** Excerpt of the Ontology Requirements Specification Document defined for our ontology

1.	Purpose
	The purpose of the TrhOnt ontology is to provide a reference model for the representation of the physiotherapy-related information that is needed for the whole physiotherapy treatment of a patient, since they step for the first time into the physiotherapist’s office, until they are discharged.
2.	Scope
	The ontology will focus on physiotherapy issues related to the glenohumeral joint.
3.	Implementation language
	The ontology has to be implemented in a formalism that allows classification of classes and realization between instances and classes.
4.	Intended Users
	∙*User 1: Physiotherapists*
5.	Intended uses
	∙*Use 1:* To record and search information about the items that compose the physiotherapy
	record of a patient.
	∙*Use 2:* To help the process of defining general treatment protocols for a specific disorder, by
	selecting the exercises that must be performed in each phase of the protocol.
	∙*Use 3* To help the process of identifying in which phase of a treatment protocol a patient is at
	some specific moment.
	∙*Use 4:* To identify which exercises are most suitable for a patient at some specific moment
	given all the information that it is known about him.
6.	Ontology requirements
	(6.a) Non-functional requirements (not applicable)
	(6.b) Functional requirements: Groups of competency questions
	∙*CQG1:* Physiotherapy record-related competency questions:
	−CQ1.1: What is the patient’s age?
	−CQ1.2: Which health issue does the patient report?
	−CQ1.3: Which are the patient’s recovery goals?
	−CQ1.4: How much pain does the patient report on the Visual Analogue Scale (VAS)?
	−CQ1.5: Which results are obtained from the exploration of the joint movement of the
	patient?
	−CQ1.6: What is the physiotherapy diagnostics of the patient?
	−CQ1.7: Which is the family and personal past history of the patient?
	−…
	∙*CQG2:* Body-related competency questions:
	−CQ2.1: Which are the body parts that compose a more general body part?
	−CQ2.2: Which is the laterality of a specific body part?
	−…
	∙*CQG3:* Treatment protocol-related competency questions:
	−CQ3.1: Which is the type of a movement?
	−CQ3.2: Which body part does a movement refer to?
	−CQ3.3: Which range of movement does a movement cover?
	−CQ3.4: Which movements compose an exercise?
	−CQ3.5: Which exercises compose a phase of a treatment protocol?
	−CQ3.6: Which are the conditions that an exercise must fulfill to be a candidate exercise for
	a phase of a treatment protocol?
	−…
	∙*CQG4:* General competency questions:
	−CQ4.1: Which are the conditions that a patient must fulfill in order to be in a phase of a
	treatment protocol?
	−CQ4.2: Which phase is a patient in?
	−CQ4.3: Which exercises are recommended for a patient at some specific moment?
	−CQ4.4: Which exercises are contraindicated for a patient at some specific moment?
	−CQ4.5: Which exercises do patients usually perform badly?
	−…
7.	Pre-glossary of terms
	Patient, goal, joint, movement, exercise, …
  Once the ORSD was generated, we performed a search for candidate knowledge resources. The search was performed following the activities defined in Scenarios 2 and 4 of NeOn, which will be explained later. The outcome of these activities were the KiReSOnt and GlenOnt ontology modules.
- *Scheduling:* The selected ontology network life cycle was the Six-Phase Waterfall, described in [22], because apart from the initiation, design, implementation and maintenance phases that 4-phase cycles usually include, it integrates a reuse phase and a re-engineering phase.
- *Conceptualization and Formalization:* Both activities were performed together to obtain a formal model of the ontology, where all the classes and properties that are needed to answer the competency questions were described by means of a Description Logic [23] (see “” section).
- *Implementation:* The formal model was implemented in the ontology language OWL 2 DL [24] using Protégé 5.0.0 [25].

### Scenario 2: reusing and re-engineering non-ontological resources

This scenario was used to select non-ontological resources that represent information related to joint movements, rehabilitation exercises and treatment protocols for disorders of the shoulder, and convert that information into one ontology. Two processes were carried out: reuse and re-engineering. The reuse process comprises three activities:

- *Search non-ontological resources:* Among others, a document about exercises and treatment protocols for rehabilitation after shoulder dislocation from the National Health Service (NHS) was found [26]. Moreover, a database of shoulder movements and exercises from a Kinect-based telerehabilitation system [27] was considered, as well as a set of treatment protocols for several shoulder-related disorders provided by expert physiotherapists. We restrict the description of the remaining activities to these resources.
- *Assess the set of candidate non-ontological resources:* We performed the assessment keeping in mind the intended uses of the target ontology (Table 2). In the case of resources that contain movements the quality of their description was assessed (i.e. does the movement indicate the initial and final position? Does it indicate the ROM?). In the case of resources that contain exercises, the quality of the description and the easiness to identify single movements within those exercises was evaluated. Finally, concerning resources that contain treatment protocols, we took into account the number of disorders that were considered, as well as the existence of phases in those protocols and conditions to classify patients in phases.

**Table 2:** Summarized assessment of candidate non-ontological resources

	NHS document	Database Kinect-based system	General treatment protocols
Movements: Quality of description	–	✓	–
Exercises: Quality of description	✓	✓	–
Exercises: Easiness to identify movements	X	✓	–
Protocols: Number of disorders	1	–	10
Protocols: Phases	✓	–	✓
Protocols: Transition conditions	X	–	✓

**Fig. 2:**
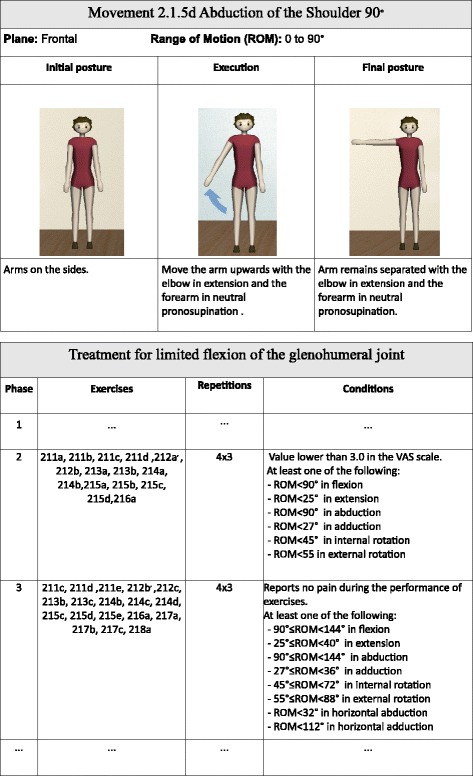
Example of movement and treatment protocol. Example of movement and excerpt of treatment protocol from the selected non-ontological resources

A tick (✓) indicates that the resource fulfils the requirement, an X that the resource does not fulfill it, and a hyphen (–) that the requirement does not apply to that resource
- *Select the most appropriate non-ontological resources:* We selected the database of the Kinect-based telerehabilitation system as a resource for movements and exercises, due to the richness of their descriptions, which provide great information for our reasoning purposes. Moreover, we selected the pool of treatment protocols provided by expert physiotherapists since it covers a wide range of disorders with definition of phases and their conditions (Fig. 2).

After the reuse process, the re-engineering process was carried out to obtain an ontology from the gathered information. Three activities were performed:

- *Non-ontological resource reverse engineering:* the resources were analyzed to identify their underlying components. In the case of movements, their name, type (flexion, extension, internal/external rotation, (horizontal) abduction, (horizontal) adduction), range of motion, plane (frontal, sagittal, transverse), initial/final posture, execution and affected body location were identified. It was also detected that in some cases a single movement is composed of more than one submovement that take place simultaneously but with different values for the {type, ROM, location} triplet. In the case of exercises their name and sequence of movements were considered. As for treatment protocols, their name, related disorder, sequence of phases (which are made up of a collection of exercises), conditions of the phases, number of repetitions of each exercise and number of times the whole phase must be repeated in the same session were identified.
- *Non-ontological resource transformation:* A conceptual model relating the underlying components identified in the previous activity was generated.
- *Ontology forward engineering:* A formal model expressed in a Description Logic was generated from the conceptual model and later implemented in OWL 2 DL using Protégé (see “” section). The resulting ontology module, KiReSOnt, was the outcome of this scenario.

### Scenario 4: reusing and re-engineering ontological resources

This scenario was used to select ontological resources that represent the glenohumeral joint and related body parts. As in the previous scenario, reuse and re-engineering were performed. More specifically, four activities were carried out in the reuse process:

- *Ontology search:* The search for an ontology that covered only the glenohumeral joint and its related body parts was unsuccessful, so we expanded the search to ontologies that cover the whole human body. Two candidate ontologies were selected: OpenGALEN [28] and FMA [7].
- *Ontology assessment:* The assessment was performed taking into account five criteria: Coverage, Understandability effort, Integration effort, Reuse economic cost and Reliability. We restricted the assessment to the Human Anatomy extension of OpenGALEN. In the case of FMA, version 4.0 was assessed (Table 3).

**Table 3:** Summarized assessment of candidate ontological resources

	Requirements	OpenGALEN	FMA
Coverage	It must cover at least the glenohumeral joint and its related body parts at great detail	✓	✓
Understandability effort	Pruning supported by a physiotherapist will be needed to obtain a module about the glenohumeral joint. Thus the structure of the ontology in ontology development tools such as Protégé must be easy to understand	Too many classes defined at the top level, it makes it difficult to understand the actual hierarchy. Many classes have very long names, which are difficult to read.	✓
Integration effort	It should be easy to integrate the candidate ontology with the ontology being developed. Moreover its implementation must adapt to the reasoner being used, and be logically satisfiable. In our case it is sufficient if the glenohumeral joint module is satisfiable.	✓	It includes unsatisfiable classes, but it is known that satisfiable modules can be obtained from it [19, 31]
Reuse economic cost	It refers to the cost of accessing and using the ontology, including licensing costs.	30 man-hours. No licensing fees.	20 man-hours. No licensing fees.
Reliability	The candidate ontology should come from reliable sources	✓	✓
- *Ontology comparison:* Both ontologies cover the domain of the glenohumeral joint to an appropriate extent. Moreover, we think that the hierarchy and nomenclature used in FMA are much clearer than those in OpenGALEN, which reduces the expected man-hours of work and thus the reuse economic cost. Since an implementation of both ontologies in OWL exists, both of them are suitable for OWL reasoners. However FMA includes unsatisfiable classes [29, 30], as opposed to OpenGALEN, although the literature has proved that fully satisfiable modules can be obtained from it [31]. Both ontologies are considered reliable since they were developed by reputable institutions and have been used in multiple projects throughout the years [32–35].
- *Ontology selection:* Given the need of involving a physiotherapist for pruning the ontology, we opted for selecting the FMA due to its clarity, always keeping in mind that we would need to check the satisfiability of the glenohumeral joint module once extracted.

After the reuse process, the re-engineering process was carried out to obtain the glenohumeral joint module. More precisely, two activities were performed:

- *Ontology re-specification:* The scope of the FMA ontology (with over 104,000 classes and 170 properties) was modified to consider just the glenohumeral joint and its related classes.
- *Ontology re-conceptualization:* We pruned the FMA ontology with the help of a module extractor [36, 37] and a physiotherapist to obtain the glenohumeral joint module, used to represent the concepts about rehabilitation processes of shoulder pathologies. The outcome was the GlenOnt ontology module (with 2054 classes and 23 properties). The module extractor works selecting concepts that are logically connected to a list of concepts passed as an argument. This way we obtained a module of classes and properties composed of elements connected between them. In our case we performed an upper hierarchy extraction using “GlenoHumeral Joint" as the only argument for the extraction process. A concept selected this way will always be connected with some other hierarchically or by a property. Then we performed a clean-up process to remove those concepts that were clearly not related with upper limbs (e.g. toe, ankle, pelvis). After that, we applied another round of the module extractor to remove “orphan" terms that might be left after the removal. Finally, this new module was presented to a physiotherapist that checked it manually, and validated its content removing those terms that were considered inadequate for the representation of upper limb pathologies in rehabilitation. This module proved to be free of unsatisfiable classes. We also incorporated UMLS (Unifided Medical Language System [38]) codes for those FMA classes that had an equivalent class in UMLS. This resulted in 272 classes from GlenOnt for which alignment axioms appeared in the UMLS repository, allowing interoperability with other sources that use this terminology.

## Results

We developed a new application ontology, named TRHONT, which imports both KIRESONT and GLENONT ontology modules, and contains other physiotherapy-related information that will be presented next. The resulting ontology covers the four intended uses mentioned in the ORSD (see Scenario 1 in “Methods”section), which are related to the competency questions listed in that same document.

### Results for intended use 1

In this intended use the ontology is regarded as a means to record and search information about the items that compose the physiotherapy record of a patient. It must be able to answer the competency questions in groups CQG1 and CQG2 (see Table 1).

The core class is PhysiotherapyRecord. Each Patient is related to their physiotherapy record(s), which is composed of a set of answers.

$$\begin{array}{@{}rcl@{}} \texttt{Patient} & \sqsubseteq & \exists\texttt{hasRecord.} \\[-5pt] &&\texttt{PhysiotherapyRecord}\\ \texttt{PhysiotherapyRecord} & \sqsubseteq & \exists\texttt{hasAnswer.Answer}\\ \end{array} $$

For each of the competency questions of CQG1 a representation of its answer was defined within the physiotherapy record. For example class CA1.4 is used to represent the answer to *“CQ1.4: How much pain does the patient report on the Visual Analogue Scale (VAS)?”*, and includes the necessary properties (hasVASvalue) to store the patient’s response as well as restrictions in its type and/or value (double[ ≥0.0, ≤10.0]). When needed, other classes related to the terms in the competency questions were defined to represent more complicated concepts (e.g. MovementExploration).

$$\begin{array}{@{}rcl@{}} \begin{aligned} &\texttt{CA1.1}\\[-2pt] & \quad \equiv \texttt{Answer} \sqcap \exists\texttt{hasAge.integer[\(\geq\)0]}\\[-3pt] &\texttt{CA1.4}\\[-2pt] &\quad \equiv\! \texttt{Answer} \sqcap \exists\texttt{hasVASvalue.double}[\!\geq\!0.0,\leq\!10.0]\\[-3pt] &\texttt{CA1.5}\\[-2pt] &\quad \equiv \exists\texttt{hasMovementExploration.}\\[-2pt] &\qquad\,\,\texttt{MovementExploration}\\[-3pt] &\texttt{MovementExploration} \\[-2pt] &\qquad \,\sqsubseteq\exists\texttt{hasMovementType.MovementType} \sqcap\\[-2pt] & \qquad \,\,\exists\texttt{hasLocation.Joint} \sqcap \exists\texttt{hasROMvalue.}\\[-2pt] &\qquad\,\,\texttt{double} \sqcap \exists\texttt{hasPain.boolean}\\[-3pt] &\texttt{MovementType} \\[-2pt] &\quad\equiv \texttt{Flexion} \sqcup \texttt{Extension} \sqcup \texttt{ExtRotation} \sqcup\\[-2pt] &\qquad\,\,\texttt{IntRotation} \sqcup \texttt{Abduction} \sqcup \texttt{Adduction} \sqcup\\[-2pt] &\qquad\,\,\texttt{HorizAbduction} \sqcup \texttt{HorizAdduction}\\[-3pt] &\texttt{CA1.7} \\[-2pt] & \quad \equiv\exists\texttt{hasPastHistory.}\\[-2pt] &\qquad\,\,\texttt{FamilyOrPersonalPastHistoryItem}\\[-3pt] &\texttt{FamilyOrPersonalPastHistoryItem}\\[-2pt] &\quad \equiv \texttt{PathologicalCondition} \sqcap \exists\texttt{hasPatient.}\\[-2pt] &\qquad\texttt{(Self} \sqcup \texttt{Relative)} \sqcap \forall\texttt{hasIntensity.}\\[-2pt] &\qquad\texttt{Intensity} \sqcap \forall\texttt{hasTimespan.Timespan}\\[-3pt] &\texttt{DislocationOfLeftGlenohumeralJoint}\\[-2pt] &\quad\sqsubseteq\texttt{PathologicalCondition} \end{aligned} \end{array} $$

**Fig. 3:**
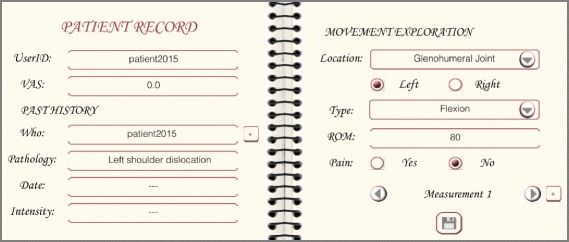
Patient record. Excerpt of the patient record of patient patient2015

Recorded answers about a specific patient are represented as individuals of classes in the ontology. Hence, the information about patient with ID patient2015 seen in Fig. 3 is transformed, among others, into the following set of triples:

$$\begin{array}{@{}rcl@{}} \langle\texttt{patient2015} & \texttt{rdf:type} & \texttt{Patient}\rangle\\[-2pt] \langle\texttt{patient2015} & \texttt{hasRecord} & \texttt{record2015}\rangle\\[-2pt] \langle\texttt{record2015} & \texttt{rdf:type} & \texttt{PhysiotherapyRecord}\rangle\\[-2pt] \langle\texttt{record2015} & \texttt{hasAnswer} & \texttt{ca1.4}\rangle\\[-2pt] \langle\texttt{ca1.4} & \texttt{rdf:type} & \texttt{CA1.4}\rangle\\[-2pt] \langle\texttt{ca1.4} & \texttt{hasVASvalue} & \texttt{0.0}\rangle\\[-2pt] \langle\texttt{record2015} & \texttt{hasAnswer} & \texttt{ca1.5}\rangle\\[-2pt] \langle\texttt{ca1.5} & \texttt{rdf:type} & \texttt{CA1.5}\rangle\\[-2pt] \langle\texttt{ca1.5} & \texttt{hasMovementExploration} & \texttt{movexp1}\rangle\\[-2pt] \langle\texttt{movexp1} & \texttt{rdf:type} & \texttt{MovementExploration}\rangle\\[-2pt] \langle\texttt{movexp1} & \texttt{hasMovementType} & \texttt{flexion}\rangle\\[-2pt] \langle\texttt{movexp1} & \texttt{hasLocation} & \texttt{leftGlenoJoint2015}\rangle\\[-2pt] \langle\texttt{leftGlenoJoint2015} & \texttt{rdf:type} & \texttt{GlenohumeralJoint}\rangle\\[-2pt] \langle\texttt{movexp1} & \texttt{hasROMvalue} & \texttt{80}\rangle\\[-2pt] \langle\texttt{movexp1} & \texttt{hasPain} & \texttt{false}\rangle\\[-2pt] \langle\texttt{record2015} & \texttt{hasAnswer} & \texttt{ca1.7}\rangle\\[-2pt] \langle\texttt{ca1.7} & \texttt{rdf:type} & \texttt{CA1.7}\rangle\\[-2pt] \langle\texttt{ca1.7} & \texttt{hasPastHistory} & \texttt{phi1}\rangle\\[-2pt] \langle\texttt{phi1} & \texttt{rdf:type} & \texttt{DislocationOfLeftGlenohumeralJoint}\rangle\\[-2pt] \langle\texttt{phi1} & \texttt{hasPatient} & \texttt{self}\rangle\\ \end{array} $$

**Fig. 4:**
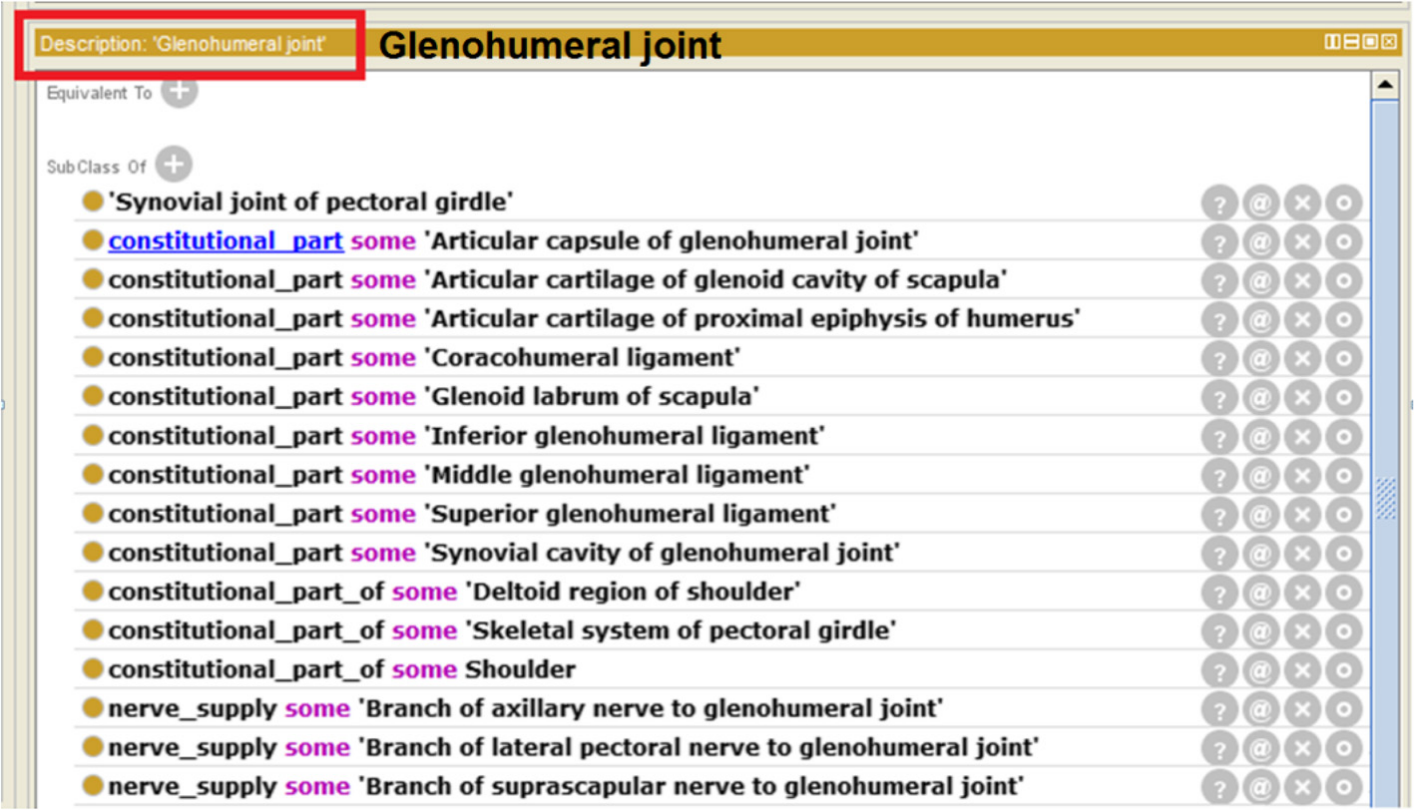
Glenohumeral joint. Glenohumeral Joint class description in Protégé

Competency questions in CQG2 can be answered by means of the GLENONT part of the ontology that was created in Scenario 4. For example, one relevant property in that ontology is constitutional_part, used to describe meronymy relationships between body parts. In Fig. 4 we show a snapshot of the class GlenohumeralJoint. It illustrates the description of this joint and how the relations with other body parts and anatomical structures are represented (e.g. constitutional_part, constitutional_part_of, nerve_supply).

### Results for intended use 2

In the second intended use the ontology is seen as a means to help the process of defining general treatment protocols for a specific disorder. It should help in the selection of the exercises that must be performed in each of the phases of the protocol and it must be able to answer the competency questions in group CQG3 (see Table 1). These requirements are covered by the definitions of the KIRESONT ontology module, which was created from non-ontological resources in Scenario 2.

#### Representation of movements, exercises and treatment protocols

A Movement is represented by its initial and final postures, and is composed of one or more Submovements that take place simultaneously within that movement. Simultaneity is needed for movements that occur in more than one anatomical plane (e.g. diagonals) or which require the movement of two joints at the same time (e.g. both right and left glenohumeral joints).

$$\begin{array}{@{}rcl@{}} \texttt{Movement} & \equiv & \exists\texttt{hasComponent.Submovement} \end{array} $$

For each Submovement its Joint, MovementType and ROM are represented, which for example can be used to answer competency questions CQ3.1 to CQ3.3

$$\begin{array}{@{}rcl@{}} \texttt{Submovement} & \sqsubseteq & \exists\texttt{hasLocation.Joint} \sqcap\\ &&\exists\texttt{hasMovementType.}\\ &&\texttt{MovementType} \sqcap \exists\texttt{hasROMmin.}\\ &&\texttt{integer} \sqcap \exists\texttt{hasROMmax.integer} \end{array} $$

Mov2.1.5d and Mov2.2.1z are examples of classes of movements with one and more submovements respectively.

$$\begin{array}{@{}rcl@{}} \begin{aligned} &\texttt{Mov2.1.5d}\\[-1pt] &\quad \equiv \texttt{Movement} \sqcap \exists\texttt{hasInitialPosture.}\\[-1pt] &\qquad\,\,\texttt{value\{`Arms on the sides'\}} \sqcap\\[-1pt] & \qquad\,\,\exists\texttt{hasFinalPosture.value\{`Arm remains}\\[-1pt] &\qquad\,\,\texttt{separated...'\}} \exists\texttt{hasComponent.}\\[-1pt] &\qquad\,\texttt{(Submovement}\sqcap \exists\texttt{hasLocation.}\\[-1pt] &\qquad\,\,\texttt{GlenohumeralJoint}\sqcap \exists\texttt{hasMovementType.}\\[-1pt] &\qquad\texttt{\,\,Abduction}\sqcap \exists\texttt{hasROMmin.value\{0\}} \sqcap \\[-1pt] &\qquad\,\,\exists\texttt{hasROMmax.value\{90\})}\\[-1pt] &\texttt{Mov2.1.5d}\\[-1pt] &\quad \sqsubseteq \exists\texttt{hasName.value\{`Abduction of the}\\[-1pt] &\qquad\,\,\texttt{shoulder at 90 degrees'\}}\\[-1pt] &\texttt{Mov2.2.1z}\\[-1pt] & \quad\equiv \texttt{Movement} \sqcap \exists\texttt{hasInitialPosture.}\\[-1pt] \end{aligned} \end{array} $$

$$\begin{array}{@{}rcl@{}} \begin{aligned} &\qquad\texttt{value\{`The initial posture for...'\}} \sqcap\\ & \qquad \exists\texttt{hasFinalPosture.value\{`Arm flexed }\\ &\qquad\texttt{and adducted...'\}} \sqcap \exists\texttt{hasComponent.}\\ &\qquad\texttt{(Submovement}\sqcap \exists\texttt{hasLocation.}\\ &\qquad\texttt{GlenohumeralJoint}\sqcap \exists\texttt{hasMovementType.}\\ &\qquad\texttt{Flexion}\sqcap \exists\texttt{hasROMmin.value\{0\}} \sqcap\\ &\qquad\exists\texttt{hasROMmax.value\{\!180\})}\! \sqcap \exists\texttt{hasComponent.}\\ &\qquad\texttt{(Submovement}\sqcap \exists\texttt{hasLocation.}\\ &\qquad\texttt{GlenohumeralJoint}\sqcap \exists\texttt{hasMovementType.}\\ &\qquad\texttt{Adduction}\sqcap \exists\texttt{hasROMmin.value\{0\}} \sqcap\\ &\qquad\exists\texttt{hasROMmax.value\{50\})} \sqcap \exists\texttt{hasComponent.}\\ &\qquad\texttt{(Submovement}\sqcap \exists\texttt{hasLocation.}\\ &\qquad\texttt{GlenohumeralJoint}\sqcap \exists\texttt{hasMovementType.}\\ &\qquad\texttt{ExtRotation}\sqcap \exists\texttt{hasROMmin.value\{0\}} \sqcap\\ &\qquad\exists\texttt{hasROMmax.value\{90\})}\\ &\texttt{Mov2.2.1z}\\ &\quad \sqsubseteq \exists\texttt{hasName.value\{`Diagonal of flexion,}\\ &\qquad\texttt{ adduction and external rotation'\}} \end{aligned} \end{array} $$

An Exercise is represented as a sequence of movements. Thus, every exercise must have an initial movement, which can be followed by another movement, and so on (This serves to answer CQ3.4). For example, in the case of Exer2.1.5d, this exercise is composed of two movements. The initial movement belongs to the class Mov2.1.5d, while the second one belongs to the class Mov2.1.5d_inv.

$$\begin{array}{@{}rcl@{}} \texttt{Exercise} & \equiv & \exists\texttt{hasMovement.Movement}\\[-1pt] \texttt{Exer2.1.5d} & \equiv & \texttt{Exercise} \sqcap \exists\texttt{hasMovement.}\\[-1pt] &&\texttt{(Mov2.1.5d} \sqcap \exists\texttt{hasMovNum.}\\[-1pt] &&\,\,\texttt{value\{1\})} \sqcap \exists\texttt{hasMovement.}\\[-1pt] &&\texttt{(Mov2.1.5d\_inv} \sqcap \exists\texttt{hasMovNum.}\\[-1pt] &&\texttt{\,\,value\{2\})} \sqcap \texttt{=2 hasMovement.}\\[-1pt] &&\,\,\texttt{Movement} \end{array} $$

A treatment protocol is represented as a sequence of phases. Each phase contains a sequence of exercises to be performed during that phase, as well as the conditions that indicate when a patient is in that phase. Actually, those conditions are the key for the conceptualization. They specify the Range Of Motion (ROM) that patients may achieve and the pain they may report during the performance of the exercises. Next, the representation of the treatment protocol for limited flexion of the glenohumeral joint shown in Fig. 2 is presented:

$$\begin{array}{@{}rcl@{}} \begin{aligned} &\texttt{TreatmentProtFlexGlenoJ}\\ &\quad \equiv \texttt{TreatmentProtocol} \sqcap \exists\texttt{hasPhase.}\\ &\qquad\texttt{(Phase1FlexGlenoJ} \sqcap \exists\texttt{hasPhaseNum.}\\ &\qquad\,\,\texttt{value\{1\})} \sqcap \exists\texttt{hasPhase.}\\ &\qquad\texttt{(Phase2FlexGlenoJ} \sqcap \exists\texttt{hasPhaseNum.}\\ &\qquad\,\,\texttt{value\{2\})} \sqcap\texttt{...}\sqcap\\ &\qquad\exists\texttt{hasPhase.(Phase5FlexGlenoJ} \sqcap\\ &\qquad\exists\texttt{hasPhaseNum.value\{5\})}\\ &\texttt{Phase2FlexGlenoJ}\\ &\quad \equiv \texttt{Phase} \sqcap \\ &\qquad\,\,\exists\texttt{hasExercise.\!\!(Exer2\!.\!1\!.1a} \sqcap \!\exists\texttt{hasExerNum.}\\ &\qquad\,\,\texttt{value\{1\})} \sqcap \exists\texttt{hasExercise.(Exer2.1.1b} \sqcap\\ &\qquad\,\,\exists\texttt{hasExerNum.value\{2\})} \sqcap \texttt{...}\sqcap\\ &\qquad\,\,\exists\texttt{hasExercise.\!\!(Exer2\!.\!1\!.\!6a} \sqcap \exists\texttt{hasExerNum.}\\ &\qquad\,\,\texttt{value\{15\})} \sqcap \exists\texttt{hasSeries.value\{4\}} \sqcap\\ &\qquad\,\,\exists\texttt{hasConditions.Cond2FlexGlenoJ}\\ &\texttt{Cond2FlexGlenoJ}\\ &\quad\equiv \exists\texttt{ROMFlex.double[\(<\)90.0]} \sqcap \exists\texttt{ROMExt.}\\ &\qquad\,\,\texttt{double[\(<\)25.0]} \sqcap \exists\texttt{ROMAbdu.double}\\ &\qquad\,\,\texttt{[\(<\)90.0]} \sqcap \exists\texttt{ROMAddu.double[\(<\)27.0]} \sqcap \\ &\qquad\,\,\exists\texttt{ROMIntRot.double[\(<\)45.0]} \sqcap \exists\texttt{ROMExtRot.}\\ &\qquad\,\,\texttt{double[\(<\)55.0]} \sqcap \exists\texttt{hasVASvalue.}\\ &\qquad\,\,\texttt{double[\(<\)3.0]} \end{aligned} \end{array} $$

It should be noticed that the set of classes of movements, exercises and protocols in KIRESONT can be extended by physiotherapists. Currently we are developing a graphical tool for this purpose (see *Context of use of TrhOnt* in “Discussion” section).

#### Selection of the exercises to be performed during a phase

Whenever a physiotherapist creates a general treatment protocol, they can rely on ontology-based reasoning to select the exercises for each phase. Once the number of phases of the protocol has been defined alongside the patient assessment conditions of each phase, new class descriptions capturing the notion of candidate exercises for each phase are automatically generated and included in the ontology. For example, class CandExe2FlexGlenoJ describes the candidate exercises for phase 2 of the protocol for patients with limited flexion of the glenohumeral joint. A candidate exercise for this phase must be composed of at least one movement that is allowed in this phase, and more importantly, all its movements must also be allowed in this phase.

$$\begin{array}{@{}rcl@{}} \begin{aligned} &\texttt{CandExe2FlexGlenoJ}\\ &\quad \equiv\texttt{Exercise} \sqcap \exists\texttt{hasMovement.}\\ &\,\ \qquad\texttt{Phase2FlexGlenoJAllowedMov} \sqcap\\ &\,\ \qquad\forall\texttt{hasMovement.Phase2Flex}\\ &\,\ \qquad\texttt{GlenoJAllowedMov}\\ &\texttt{Phase2FlexGlenoJAllowedMov}\\ &\quad \equiv \texttt{Movement} \sqcap \texttt{(MovAbduGJLessEqual90} \sqcup\\ &\,\ \qquad\texttt{MovAdduGJLessEqual27} \sqcup\\ &\,\ \qquad\texttt{MovExtGJLessEqual25} \sqcup\\ &\,\ \qquad\texttt{MovExtRotGJLessEqual55} \sqcup \\ &\,\ \qquad\texttt{MovFlexGJLessEqual90} \sqcup\\ &\,\ \qquad\texttt{MovIntRotGJLessEqual45)} \end{aligned} \end{array} $$

A movement is allowed in a phase if it complies with the conditions of the phase. As can be seen in the definition of CandExe2FlexGlenoJ, the movements allowed in phase 2 of the protocol for the limited flexion of the glenohumeral join must belong to the classes MovAbduGJLessEqual90, MovAdduGJLessEqual27, MovExtGJLessEqual25, MovExtRotGJLessEqual55, MovFlexGJLessEqual90 or MovIntRotGJLessEqual45. For example, MovFlexGJLessEqual90 represents those movements of flexion of the glenohumeral joint with a ROM lower or equal to 90°.

$$\begin{array}{@{}rcl@{}} \begin{aligned} &\texttt{MovFlexGJLessEqual90}\\ &\quad \equiv \texttt{Movement} \sqcap \exists\texttt{hasComponent.(Submovement}\\ &\qquad\sqcap \exists\texttt{hasLocation.} \texttt{GlenohumeralJoint} \sqcap\\ &\qquad \exists\texttt{hasMovementType.Flexion} \sqcap \exists\texttt{hasROMmax.}\\ &\qquad\texttt{double[\(\leq\)90.0])} \end{aligned} \end{array} $$

Specific movements (e.g. Mov2.1.5d, Mov2.2.1z) are properly classified as subclasses of these sort of class descriptions (e.g. Mov2.1.5d is classified as subclass of MovAbduGJLessEqual90), and moreover, exercises get classified as subclasses of the corresponding candidate classes (e.g CandExe2FlexGlenoJ), depending on the movements they include. More precisely, any exercise class that only contains movements that are subclasses of Phase2FlexGlenoJAllowedMov is classified as a subclass of CandExe2FlexGlenoJ, and will be presented to the physiotherapist on demand of exercises for phase 2 of the selected protocol. This happens, for instance, with Exer2.1.5d.

If they decide to select that exercise class for the protocol definition, the following new axiom is created:

$$\begin{array}{@{}rcl@{}} \texttt{Exer2.1.5d} & \sqsubseteq & \texttt{Exe2FlexGlenoJ} \end{array} $$

Now, Exer2.1.5d will not only be a subclass of the class for representing candidate exercises for phase 2 (CandExe2FlexGlenoJ), but also a subclass of the class for representing proper exercises for phase 2 (Exe2FlexGlenoJ).

Classes for representing candidate exercises for other phases are defined likewise:

$$\begin{array}{@{}rcl@{}} \begin{aligned} &\texttt{CandExe3FlexGlenoJ}\\ &\quad \equiv \texttt{Exercise} \sqcap \exists\texttt{hasMovement.}\\ &\qquad\,\,\texttt{Phase3FlexGlenoJAllowedMov} \sqcap\\ &\qquad\,\,\forall\texttt{hasMovement.}\\ &\qquad\,\,\texttt{Phase3FlexGlenoJAllowedMov}\\ &\texttt{Phase3FlexGlenoJAllowedMov}\\ &\quad \equiv \texttt{Movement} \sqcap\!\! \texttt{(MovAbduGJLessEqual144} \sqcup\\ &\qquad\,\,\texttt{MovAdduGJLessEqual36} \sqcup \\ &\qquad\,\,\texttt{MovExtGJLessEqual40} \sqcup\\ &\qquad\,\,\texttt{MovExtRotGJLessEqual88} \sqcup \\ &\qquad\,\,\texttt{MovFlexGJLessEqual144} \sqcup\\ &\qquad\,\,\texttt{MovIntRotGJLessEqual72} \sqcup\\ &\qquad\,\,\texttt{MovHorAbduGJLessEqual32} \sqcup\\ &\qquad\,\,\texttt{MovHorAdduGJLessEqual112)}\\ \end{aligned} \end{array} $$

Notice that one of the classes (CandExe3FlexGlenoJ) subsumes the other (CandExe2FlexGlenoJ), meaning that all the exercises classified in CandExe2FlexGlenoJ are also members of CandExe3FlexGlenoJ. This is considered conceptually correct by physiotherapists, because at any point they should be able to select milder exercises, in order, for example, to warm the joint up.

### Results for intended use 3

The third intended use gives response to some of the competency questions defined in CQG4 (see Table 1). The ontology is used as an artifact to help the process of identifying in which phase of a treatment protocol a patient is at some specific moment. This is done by taking into account the results of the movement capability explorations of the patient at that time. Analogously to the previous intended use 2, the classification is guided by the conditions specified in the phases of the protocols. In this case, conditions regarding the ROM and the pain are considered. Then, one ontology class is automatically created for each phase of each protocol based on the associated conditions. For example, the definitions of the classes Patient2FlexGlenoJ and Patient3FlexGlenoJ that can be seen next represent those patients who are respectively in phase 2 and 3 of the protocol to treat the limited flexion of the shoulder.

$$\begin{array}{@{}rcl@{}} \begin{aligned} &\texttt{Patient2FlexGlenoJ}\\[-1pt] &\quad \equiv \texttt{Patient} \sqcap \exists\texttt{hasRecord.}\\[-1pt] &\qquad\texttt{(PhysiotherapyRecord} \sqcap \exists\texttt{hasAnswer.}\\[-1pt] &\qquad\texttt{(CA1.4} \sqcap \exists\texttt{hasVASvalue.double[\(<\)3.0])} \sqcap\\[-1pt] &\qquad\,\,\exists\texttt{hasAnswer.(CA1.5} \sqcap \\[-1pt] &\qquad\,\,\exists\texttt{hasMovementExploration.}\\[-1pt] &\qquad\texttt{(MovExploFlexGJLessThan90} \sqcup\\[-1pt] &\qquad\,\,\texttt{MovExploExtGJLessThan25} \sqcup\\[-1pt] &\qquad\,\, \texttt{MovExploAbduGJLessThan90} \sqcup\\[-1pt] &\qquad\,\, \texttt{MovExploAdduGJLessThan27} \sqcup \\[-1pt] &\qquad\,\,\texttt{MovExploIntRotGJLessThan45} \sqcup\\[-1pt] &\qquad\,\, \texttt{MovExploExtRotGJLessThan55)))}\\[-1pt] &\texttt{Patient3FlexGlenoJ}\\[-1pt] &\quad \equiv \texttt{Patient} \sqcap \exists\texttt{hasRecord.}\\[-1pt] &\qquad\texttt{(PhysiotherapyRecord} \sqcap \exists\texttt{hasAnswer.}\\[-1pt] &\qquad \texttt{(CA1.5} \sqcap \exists\texttt{hasMovementExploration.}\\[-1pt] &\qquad\texttt{((MovExploFlexGJBetween90And143} \sqcup \\[-1pt] &\qquad\,\,\texttt{MovExploExtGJBetween25And39} \sqcup\\[-1pt] &\qquad\,\,\texttt{MovExploAbduGJBetween90And143} \sqcup \\[-1pt] &\qquad\,\,\texttt{MovExploAdduGJBetween27And35} \sqcup\\[-1pt] &\qquad\,\,\texttt{MovExploIntRotGJBetween45And71} \sqcup \\[-1pt] &\qquad\,\, \texttt{MovExploExtRotGJBetween55And87} \sqcup\\[-1pt] &\qquad\,\,\texttt{MovExploHorAbduGJLessThan32} \sqcup\\[-1pt] &\qquad\,\,\texttt{MovExploHorAdduGJLessThan112)} \sqcap\\[-1pt] &\qquad\exists\texttt{hasPain.value\{false\})))} \end{aligned} \end{array} $$

Definitions of the classes with the prefix MovExplo* refer to one type of movement exploration assessed in a patient. For instance the definition of MovExploFlexGJLessThan90 describes an exploration of the flexion of the shoulder where the ROM achieved by the patient is below 90°.

$$\begin{array}{@{}rcl@{}} \begin{aligned} &\texttt{MovExploFlexGJLessThan90}\\ &\quad \equiv \texttt{MovementExploration} \sqcap \exists\texttt{hasLocation.}\\ &\qquad\,\,\texttt{GlenohumeralJoint} \sqcap \exists\texttt{hasMovementType.}\\ &\qquad\,\,\texttt{Flexion}\sqcap \exists\texttt{hasROMmax.double[\(<\)90.0]} \end{aligned} \end{array} $$

The other explorations are defined likewise. Thus, whenever a patient presents a movement exploration that satisfies the definition of any of the MovExplo* classes in Patient2FlexGlenoJ and reports a VAS value lower than 3.0, the patient will be classified as belonging to the class Patient2FlexGlenoJ.

For instance, considering the set of the triples about patient patient2015 presented in “Results for intended use 1” section, patient patient2015 would be classified as a Patient2FlexGlenoJ, because they have reported a VAS value of 0.0 (<3.0) and there exists in their current physiotherapy record a movement exploration of flexion of the glenohumeral joint where they achieved a ROM of 80° (which satisfies conditions of the class MovExploFlexGJLessThan90). Notice that the classification of the patient evolves alongside their evolution in the therapy: if after being in phase 2 and performing the exercises recommended for that phase the aforementioned ROM increases to 100° and the patient reports no pain when performing those exercises, some triple assertions are deleted and some others added. As a result, the patient would no longer be classified as a patient of phase 2, but as a patient of phase 3 (see previous definition for Patient3FlexGlenoJ).

### Results for intended use 4

In the last intended use, the ontology is regarded as a means to identify which exercises are most suitable for a patient at some specific moment given all the information that is known about them. Three cases are considered:

- *Recommended exercises due to the physical state of the patient:* This is done by taking into account the results of the movement explorations of the patient at that time. For example, if the movement explorations of patient2015 indicate that they are in phase 2 (intended use 3) then they have as recommended exercises those for the patients in phase 2 (that group of patients is represented by class Patient2FlexGlenoJ). Then, the following axiom represents that knowledge:
  $$\begin{array}{@{}rcl@{}} \texttt{Patient2FlexGlenoJ} &\sqsubseteq & \exists\texttt{recommended.}\\ &&\texttt{Exer2FlexGlenoJ} \end{array} $$
  Notice that the exercises of a certain phase were inferred as shown in the intended use 2.
- *Recommended/Contraindicated exercises due to general physiotherapy knowledge:* Some domain specific axioms have been added to the ontology to represent general physiotherapy knowledge such as *“People with a personal past history of dislocation of glenohumeral joint should not perform exercises that contain abduction movements with a ROM greater than 80°”* (e.g. class axioms for the left glenohumeral joint are shown in the following).
  $$\begin{array}{@{}rcl@{}} \begin{aligned} &\texttt{PatientPastDislocationLeftGlenoJ}\\[-1pt] &\quad\equiv \texttt{Patient} \sqcap \exists\texttt{hasRecord.}\\[-1pt] &\qquad\texttt{(PhysiotherapyRecord}\sqcap \\[-1pt] &\qquad\exists\texttt{hasAnswer.(CA1.7} \sqcap\\[-1pt] & \qquad\exists\texttt{hasPastHistory.}\\[-1pt] &\qquad\texttt{(DislocationOfLeftGlenoJ}\ \sqcap\\[-1pt] &\qquad\exists\texttt{hasPatient.Self)))}\\[-1pt] &\texttt{PatientPastDislocationLeftGlenoJ}\\[-1pt] &\quad\sqsubseteq \exists\texttt{contraindicated.}\\[-1pt] &\qquad\texttt{ExerAbduLeftGlenoJGreaterThan80} \end{aligned} \end{array} $$
- *Recommended/Contraindicated exercises for a specific patient:* The physiotherapist can specify at any time that an exercise is recommended/contraindicated for a specific patient. For example *“patient2015 should not perform exercises that contain extension movements”*.
  $$\begin{array}{@{}rcl@{}} {}\begin{aligned} \texttt{Patient2015} &\equiv \{\texttt{patient2015}\}\\ \texttt{Patient2015} &\sqsubseteq \exists\texttt{contraindicated.}\\ &\quad\,\,\texttt{ExerExtension}\\ \texttt{ExerExtension} &\equiv \texttt{Exercise}\sqcap\exists\texttt{hasMovement.}\\ &\quad\,\,\texttt{MovExtension}\\ \texttt{MovExtension} &\equiv \texttt{Movement}\ \sqcap\exists\texttt{hasComponent.}\\ &\quad\texttt{(Submovement} \sqcap\\ &\quad\,\,\exists\texttt{hasMovementType.Extension)} \end{aligned} \end{array} $$

Object properties recommended and contraindicated have been created to represent this knowledge.

The most suitable exercises for a patient *p* will be represented by the named classes *X*_*p*_ such that *X*_*p*_∈*R* *e* *c* *o* *m* *m* *e* *n* *d* *e* *d* *F* *o* *r*(*p*) but *X*_*p*_∉*C* *o* *n* *t* *r* *a* *i* *n* *d* *i* *c* *a* *t* *e* *d* *F* *o* *r*(*p*) where

$$\begin{array}{@{}rcl@{}} \begin{aligned} RecommendedFor(p) &= \{Z|\ namedClass(Z)\ \wedge\\ &\quad\exists Y\ \exists C (namedClass(Y)\ \wedge\\ &\quad namedClass(C)\ \wedge Z\sqsubseteq Y\ \wedge\\ &\quad p\!\in \!C \wedge C\sqsubseteq\exists recommended.Y)\}\\ ContraindicatedFor(p) &= \{Z|\ namedClass(Z)\ \wedge\\ &\quad \exists Y\ \exists C (namedClass(Y)\ \wedge\\ &\quad namedClass(C)\ \wedge Z\sqsubseteq Y\ \wedge\\ &\quad p\!\in\! C\ \!\wedge\! C\!\sqsubseteq\!\exists contraindicated.Y)\} \end{aligned} \end{array} $$

## Evaluation

In this section we present a threefold evaluation of our ontology. First, we show the results of checking our ontology using the OntOlogy Pitfall Scanner OOPS! [39] in order to diagnose potential design errors. Then, an evaluation of the ontology using criteria related to ontology quality is presented. Finally, a list of several ontology metrics regarding its size and composition is shown.

### Detection of potential pitfalls

OOPS! evaluates an ontology against a catalogue of 41 potential pitfalls classified in three levels (critical, important, minor). We performed an evaluation of our ontology and corrected the reported pitfalls. As a result, we obtained the fixed current version of the ontology. However we feel the need to introduce here some of the pitfalls that were related to the GLENONT module, because these piftalls are also present in FMA ontology. Tables 4 and 5 respectively present the critical and important pitfalls initially reported by OOPS!. For each pitfall we indicate its code, description, where in the ontology it appears, the reason why the pitfall is flagged and other useful information that is needed to understand it, its implications and how we corrected it.

**Table 4:** Critical pitfalls

Code	P28: Defining wrong symmetric relationships
Description	A relationship is defined as symmetric when the relationship is not necessarily symmetric.
Appears in	SymmetricProperty(continuous_with)
Reason	the domain of continuous_with is different from the range of continuous_with (‘Material Anatomical Entity’ vs. ‘Physical Anatomical Entity’).
Other useful information	subClassOf(‘Material Anatomical Entity’, ‘Physical Anatomical Entity’)
Implications	Let ‘Material Anatomical Entity’(x), ‘Physical Anatomical Entity’(y), continuous_with(x,y). Due to SymmetricProperty(continuous_with), the reasoner infers that ‘Physical Anatomical Entity’(x) and ‘Material Anatomical Entity’(y)
Correction	Change the domain of continuous_with to ‘Material Anatomical Entity’.
Code	P05: Defining wrong inverse relationships
Description	Two relationships are defined as inverse relationships when they are not necessarily inverse.
Appears in	inverseOf(continuous_with,continuous_with)
Reason	the domain of continuous_with is different from the range of continuous_with (‘Material Anatomical Entity’ vs. ‘Physical Anatomical Entity’).
Implications	Let ‘Material Anatomical Entity’(x), ‘Physical Anatomical Entity’(y), continuous_with(x,y). Due to inverseOf(continuous_with,continuous_with), the reasoner infers that ‘Physical Anatomical Entity’(x) and ‘Material Anatomical Entity’(y)
Correction	This pitfall corrects itself as a result of correcting pitfall P25 (see Table 5)

**Table 5:** Important pitfalls

Code	P11: Missing domain or range in properties
Description	Object and/or datatype properties without domain or range (or none of them) are included in the ontology.
Appears in	For example: http://purl.org/sig/ont/fma/part
Reason	All the cases refer to meronimy relations that can be applied to any of the classes of the ontology.
Other useful information	subClassOf(‘Physical Anatomical Entity’, ‘Material Anatomical Entity’)
Implications	None.
Correction	We chose not to change anything, since it is not an error per se, just an implication of the current domain.
Code	P25: Defining a relationship as inverse to itself.
Description	A relationship is defined as inverse of itself.
Appears in	inverseOf(continuous_with, continuous_with), inverseOf(articulates_with, articulates_with)
Reason	This relationship could have been defined as owl:SymmetricProperty instead.
Correction	Remove both inverseOf axioms. SymmetricProperty(continuous_with) and SymmetricProperty(articulates_with)already existed in the ontology.
Code	P26: Defining inverse relationships for a symmetric one.
Description	A symmetric object property is defined as inverse of another object property.
Appears in	inverseOf(continuous_with, continuous_with), SymmetricProperty(continuous_with), inverseOf(articulates_with, articulates_with), SymmetricProperty(articulates_with)
Correction	This pitfall corrects itself as a result of correcting pitfall P25.
Code	P24: Using recursive definitions.
Description	An ontology element is used in its own definition.
Appears in	continuous_with, articulates_with,‘Frontal part of head’
Other useful information	‘Frontal part of head’ $\sqsubseteq \exists $ attributed_part.( ∃(related_part.‘Frontal part of head’) ⊓(1 partition.{‘Partition’}))
Correction	The problems concerning continuous_with and articulates_with correct themselves as a result of correcting pitfall P25. Moreover, we feel that the aforementioned axiom involving ‘Frontal part of head’ is correct, so we chose not to change it.
Code	P34: Untyped class.
Description	An ontology element is used as a class without having been explicitly declared as such using the primitives owl:Class or rdfs:Class.
Appears in	‘Anatomical entity’
Correction	owl:Class(‘Anatomical entity’) added to the ontology.
Code	P30: Equivalent classes not explicitly declared.
Description	Missing the definition of equivalent classes (owl:equivalentClass) in case of duplicated concepts.
Appears in	Cheek vs. Face, Ear vs. Pinna, Mouth vs. Lip, Limb vs. Arm
Reason	The names of both classes appear in a common synset (set of synonyms) in WordNet [45].
Correction	None. We checked each of the suggestions by looking up in WordNet the synsets where each pair appears. For example, Cheek and Face appear in a synset with terms such as *Boldness*, *Nerve* and *Brass*, refering to *impudent aggressiveness*, not the body part, which is the meaning intended in the ontology. Same applies to the other pairs. Thus, we did not change anything in the ontology

### Ontology quality

Next we evaluate our ontology against several quality criteria described in [40], which are presented as part of a common framework for aspects of ontology evaluation. **Accuracy:** The definitions and descriptions in the ontology agree with the expert’s knowledge about the domain. The GLENONT ontology module was obtained from the well-known FMA ontology. The KIRESONT module and the information regarding patients were developed using actual physiotherapy records and recovery protocols, and with the support of physiotherapists. **Adaptability:** We have opted for implementing the ontology in several modules that are related to each other via import clauses. The file *GlenOnt.owl* ^1^ contains the GLENONT ontology module. The KIRESONT ontology module has been divided into two files: *KiReSOntFM.owl* ^2^, which contains generic classes and properties for describing movements, exercises and protocols, and *KiReSOnt.owl* ^3^, which contains the descriptions of specific movements, exercises and protocols (e.g. Mov2.1.5d). Finally, the main file *TrhOnt.owl* ^4^, incorporates the patient record, general axioms about physiotherapy and the relations to the other files. This choice enhances extensibility and reusability, and makes the ontology be easily adaptable to several contexts. Moreover, we provide a merged file^5^ with all the resources. **Clarity:** All the terms in the ontology have been given a non-ambiguous label or description using rdfs:label or rdfs:comment, so that the ontology communicates effectively the intended meaning of those terms. For example, class CA1.4 has been described as *Answer to the question “How much pain does the patient report on the Visual Analogue Scale (VAS)?”*, while class Mov2.1.5e has been described as *Movement of abduction of the shoulder at 90 degrees*. **Completeness:** This feature measures whether the ontology can answer all the questions that it should be able to answer, that is, how well the ontology represents the domain it models. Those questions were specified in the ORSD and it has been checked carefully that all of them can be answered. **Computational efficiency:** It must be admitted that the GLENONT module as a whole is still too big for some of our purposes. Current DL reasoners are not able to handle it in what we consider reasonable time^6^. However, we must distinguish two uses of GLENONT: when the physiotherapist is defining new movements, exercises or protocols they would have the whole GLENONT module at their disposal, so that they can choose from a wide range of body parts, because in this case the purpose of GLENONT is annotation and not reasoning. Thus, computational efficiency will not be an issue in this case. Once the definitions have been made, the handful of the classes of GLENONT that are used within them can be used as seeds in the module extractor in order to obtain on the spot a lighter module to be used in those moments where reasoning is necessary (e.g when asking for the exercises that are recommended for a patient). We are currently working on a tool that given a set of treatment protocols, automatically reduces the number of terms in the GLENONT module by using the module extractor and the terms used to describe those protocols, in such a way that no semantic loss is involved. **Conciseness:** Since the development of the ontology was made with the help of physiotherapists, we asume that the ontology does not contain irrelevant terms with regard to the domain that is being covered. Moveover, checking our ontology with OOPS! has discarded the presence of redundant terms (see pitfall P30 in Table 5). **Consistency:** Reasoning was performed on the ontology using Fact++. No inconsistencies were found.

### Ontology metrics

In Table 6 a summary of ontology metrics obtained from the Protégé development framework can be found. These metrics are related to the size of the ontology and its components.

**Table 6:** Ontology metrics

Metrics	Axiom	28181
	Logical axiom count	6161
	Class count	2351
	Object property count	65
	Data property count	35
	Individual count	134
	DL expressivity	ALCROIQ(D)
Class axioms	SubClassOf	4982
	EquivalentClasses	216
	DisjointClasses	617
	GCI count	0
	Hidden GCI count	199
Object property axioms	SubObjectPropertyOf	7
	EquivalentObjectProperties	0
	InverseObjectProperties	5
	DisjointObjectProperties	0
	FunctionalObjectProperty	0
	InverseFunctionalObjectProperty	0
	TransitiveObjectProperty	0
	SymmetricObjectProperty	2
	AsymmetricObjectProperty	0
	ReflexiveObjectProperties	0
	IrreflexiveObjectProperty	0
	ObjectPropertyDomain	49
	ObjectPropertyRange	52
	SubPropertyChainOf	3
Data property axioms	SubDataPropertyOf	0
	EquivalentDataProperties	0
	DisjointDataProperties	0
	FunctionalDataProperty	9
	DataPropertyDomain	29
	DataPropertyRange	35
Individual axioms	ClassAssertion	143
	ObjectPropertyAssertion	10
	DataPropertyAssertion	2
	NegativeObjectPropertyAssertion	0
	NegativeDataPropertyAssertion	0
	SameIndividual	0
	DifferentIndividuals	0
Annotation axioms	AnnotationAssertion	19402
	AnnotationPropertyDomain	0
	AnnotationPropertyRangeOf	0

## Discussion

TRHONT is an application ontology that can assist physiotherapists in the management of the patients’ evolution via reasoning supported by semantic technology. We can find in the literature many ontologies (e.g. [42–44]) that have been built with the purpose of supporting a precise and comprehensive semantic annotation of resources. However, TRHONT goes an step further and it also provides a framework where the reasoning process takes a relevant role.

TRHONT contains terms from the well-known FMA ontology, that covers the whole anatomical structure of the human body, as well as terms that describe the physiotherapy records of patients, and also movements of body parts, exercises for physiotherapy and treatment protocols. The selection of terms was made with the support of physiotherapists, and their descriptions turn TRHONT into an actionable tool for physioterapists in their daily work. Moreover, UMLS codes have been also incorporated to some terms imported from the FMA ontology, favouring in this way interoperability with other sources that use this terminology. TRHONT is still in active development, and we expect it to grow considerably, in the area of physiotherapy; however, in the current state it contains enough terms for supporting open contributions from professionals desiring to populate the ontology with more therapy elements. That is to say, TRHONT is ready to be used.

Next, some of the decisions made during the development of TRHONT are presented. Moreover, the scope and context of use of the ontology are also discussed.

### Decisions in the development of TRHONT

During the development process, different alternatives have been considered, and we present in the following some of the choices we made:

#### Representation of the physiotherapy record

The core class in the representation of the physiotherapy record of a patient is PhysiotherapyRecord. This class is related to Answer, whose subclasses (e.g CA1.1) represent the answers to competency questions about the physiotherapy record (see “Results for intended use 1” section). This representation facilitates the extensibility of the model, since the addition of new competency questions related to the physiotherapy record will not interfere with the current ones.

#### Storage of patient information

As we indicated in “Results for intended use 1” section, recorded answers about a specific patient are represented as individuals of classes in the ontology. However, having all the information about all the patients always stored in the assertional box of the ontology would take an unnecessary toll in the efficiency of any EHR system that uses the ontology. Thus, we envisage a use of the ontology where the patient information and the terminology part of the ontology are kept separate. Whenever a physiotherapist is treating a patient, only the information of that patient is loaded onto the ontology, and then discharged when the interaction is over. Moreover, specific classes such as Patient2015 (see “Results for intended use 4” section) are also temporary. They will be created when reasoning about the recommended/contraindicated exercises for a patient and deleted once the reasoning process is over.

#### Representation of movements, exercises and treatment protocols

In order to select the right descriptions of those core terms in the physioterapy treatments, we collaborated closely with physiotherapists. The idea was to get descriptions that mimic their conception of the elements related to the treatments. Therefore, a movement is represented by its initial and final postures, and is composed of one or more submovements that take place simultaneously within that movement. An exercise is represented as a sequence of movements, and a treatment protocol is represented as a sequence of phases. Each phase contains a sequence of exercises to be performed during that phase, as well as the conditions that indicate when a patient is in that phase. The sequential nature of the descriptions allows checking proper concatenation of them.

### Scope of TRHONT

Although in this paper we have focused on the rehabilitation of the glenohumeral joint, the exposed method can be easily reproduced in order to cover other body structures subject to rehabilitation. The same module extractor that was used as a basis in the creation of GLENONT can be used to perform hierarchical extractions of concepts using other body structures as argument for the extraction process. Other parts of TRHONT (e.g. the patient record) will not be affected by a change in the selected body structure. The scaffolding of the phases, exercises and movements can be reused. Specific information to that body structure will have to be added (e.g. if instead of the glenohumeral joint the ankle is selected, then specific exercises for the recovery of the ankle will have to be defined).

### Context of use of TRHONT

TRHONT is a nuclear part of a telerehabilitation system called KiReS, a Kinect-based system which covers, on the one hand, the needs of physiotherapists in the process of creating, designing, managing and assigning physiotherapy treatment protocols, as well as evaluating the performance of patients in those protocols; and, on the other hand, the needs of the patients, by providing them an intuitive and encouraging interface for performing exercises, which also gives useful feedback to enhance the rehabilitation process.

Most technical details related to the creation of classes and the behavior of TRHONT are encapsulated in KiReS. Its interface handles aspects concerning, among others, the creation of exercises and the design of protocols. In order to be able to design adequate protocols for the patients, first of all, exercises must be created in KiReS. Postures and movements, basic components of the exercises, are recorded by being performed in front of Kinect. Later, they are combined to create exercises. The interface provides step by step assistance in this process. Next, we present two snapshots that show respectively the appearance of the interface for recording movements and creating exercises.

**Fig. 5:**
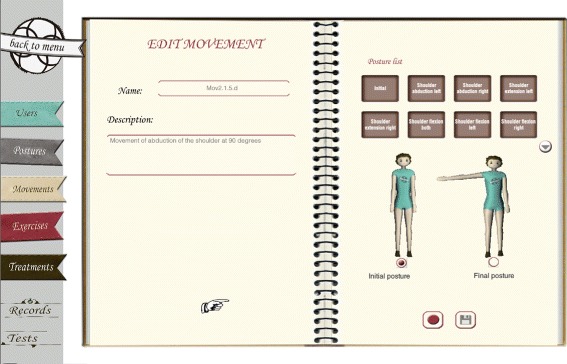
Movement recording. Interface for recording new movements in KiReS

In Fig. 5 the interface for recording new movements is shown. The definition of a movement requires, at least, to assign a name that identifies the movement and to select the initial and final postures that the movement will have. Then, the physiotherapist can visualize in the interface two avatars that show these postures and proceed to record the transition between the postures that best mimics the optimal execution of the movement. After reaching the final posture, a recording player tool is available and the physiotherapists can replay the movement and decide whether to store it or not.

**Fig. 6:**
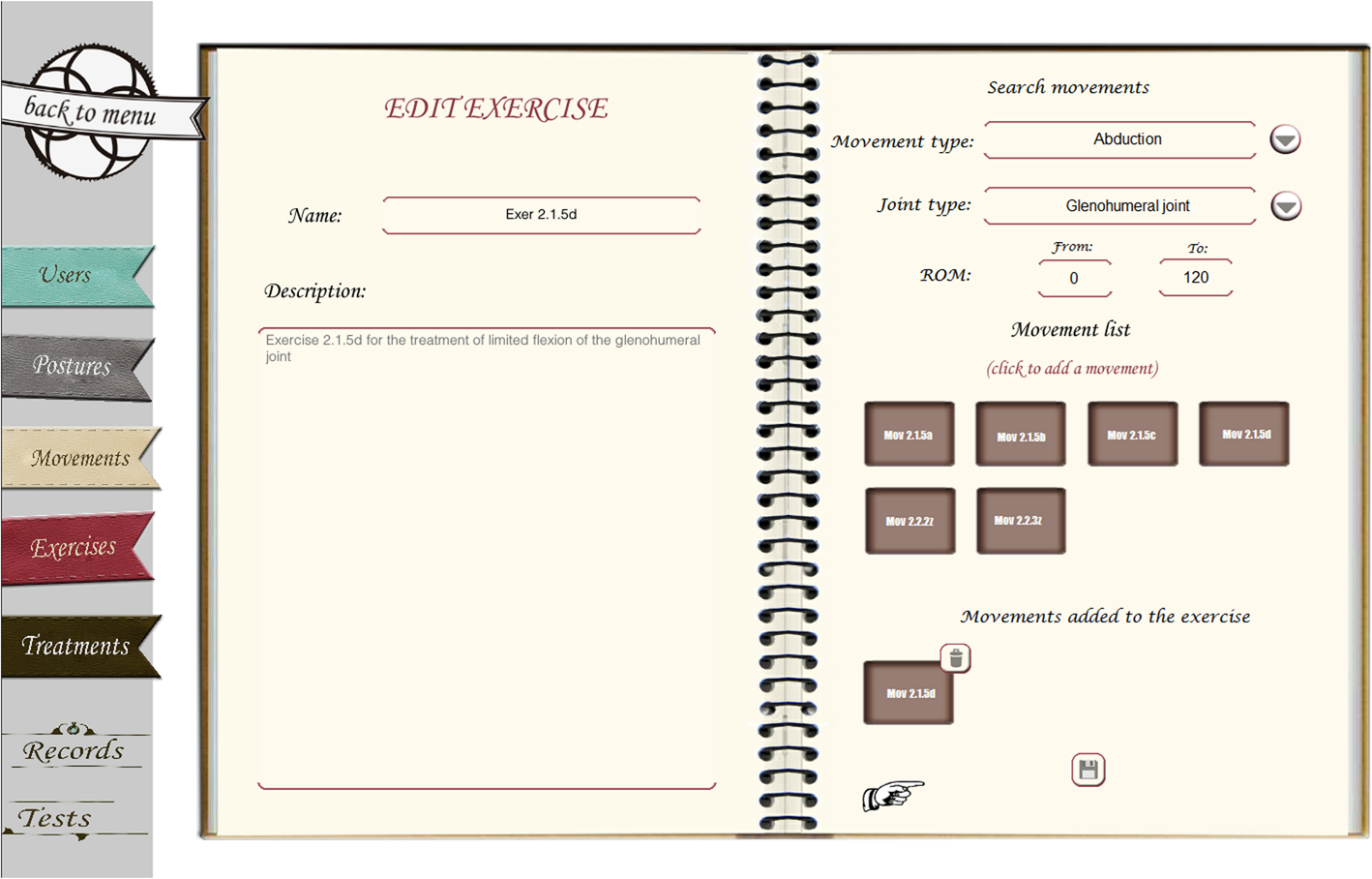
Creation of exercises. Interface for creating new exercises in KiReS

Moreover, exercises are created by a combination of one or more movements. The only restriction when combining movements is that the final posture of a movement must match the initial posture of the next one. The interface for creating exercises (see Fig. 6) allows physiotherapists to define the composition of exercises. In the left side of the interface the name and description of the exercise can be filled. The right side of the interface is divided into three areas. In the top area physiotherapists can restrict the search for movements by indicating certain conditions about them, such as the type of movement, the specific joint or the ROM. The search is performed over the list of movements stored in the system and by means of DL Queries that are transparent to the users. The area in the middle shows the results of the search (e.g. Mov2.1.5a, Mov2.1.5b,...). Finally, the last area shows the movements selected by physiotherapists (e.g. Mov2.1.5d). When selecting a movement the system checks whether the final posture of the previous movement matches the initial of the new one. If postures match, then the movement is added to the exercise. Once this is done the exercise will be stored in the system and will be available to be added to a treatment protocol.

Currently we are developing a new functionality for KiReS that will allow physiotherapists to create their own treatment protocols for their patients, guided by the ontology, using the exercises that have been stored with the aforementioned interfaces.

Movements, exercises and treatment protocols created in this way are represented internally as classes of the TRHONT ontology. This representation is generated automatically at the same time that the physiotherapists create them with KiReS.

**Fig. 7:**
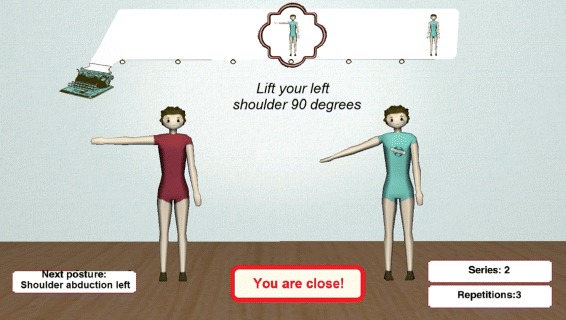
Execution of exercises. Inferface for patients when executing exercises in KiReS

Furthermore, once treatment protocols are assigned to patients, those patients are monitored at the same time they are performing the exercises for each phase of treatment (see Fig. 7. More technical details in [27]). All captured data are recorded in the KiReS database and asserted as facts in the TRHONT ontology. Due to a reasoning process (see “Results for intended use 3” section), the physiotherapists can see if the patients have overcome each phase of the recommended treatment and therefore decide whether to end the rehabilitation process or to assign new exercises to the patients.

## Conclusions

Semantic technologies have been widely used in several medical fields in order to facilitate the work of physicians. In this paper we have presented an ontology for physiotherapists from two different perspectives. On the one hand, from the point of view of its creation, by showing how it has been created by integrating information from different resources: pre-existing ontologies, databases of movements, exercises and treatment protocols, experts’ knowledge, patient records, etc. On the other hand, from the perspective of its usage and the relevant information that it provides for the physiotherapists via reasoning processes. This information includes recommended exercises depending on the physical state of the patient, the patient’s past history, etc. That is, information that can improve rehabilitation processes.

In summary, TRHONT conceptualizes medical knowledge (in order to deal with aspects related to physiotherapy), process knowledge (in order to describe treatment protocols), and instrumental knowledge (in order to describe treatment elements such as exercises). It has been designed to assist physiotherapists in several daily tasks such as recording and searching information in the physiotherapy record of a patient, defining treatment protocols by selecting suitable exercises for each phase of a protocol, determining the current state of a patient and showing their evolution, by identifying which phase of a protocol the patient is in, and detecting which exercises are most suitable for a patient at some specific moment taking into account all the information that it is known about them. TRHONT has been developed in such a way that it can be easily extended, e.g. by adding new competency questions to the physiotherapy record of the patient, or by adding new exercises or treatment protocols. Moreover, it is also reusable, since it has been implemented in several modules that could be used for other purposes. The work has been completed with a threefold evaluation of the ontology centered on piftfall detection, quality assessment and ontology metrics.

## Endnotes

^1^ Available from http://bdi.si.ehu.es/bdi/ontologies/GlenOnt.

^2^ Available from http://bdi.si.ehu.es/bdi/ontologies/KiReSOntFM.

^3^ Available from http://bdi.si.ehu.es/bdi/ontologies/KiReSOnt.

^4^ Available from http://bdi.si.ehu.es/bdi/ontologies/TrhOnt.

^5^ Available from http://bdi.si.ehu.es/bdi/ontologies/MergedTrhOnt.

^6^ More precisely, it took the FaCT++ reasoner [41] about 1.5 min to classify TRHONT in an Intel(R) Core(TM) i7-4610M CPU @ 3.00 GHz with 8 GB of RAM when the GLENONT module was used as a whole.
